# A Prospective Study Evaluating the Effects of a Nutritional Supplement Intervention on Cognition, Mood States, and Mental Performance in Video Gamers

**DOI:** 10.3390/nu11102326

**Published:** 2019-10-01

**Authors:** Jaime L. Tartar, Douglas Kalman, Susan Hewlings

**Affiliations:** 1 College of Psychology, Nova Southeastern University, Davie, FL 33314 1, USA; tartar@nova.edu; 2 College of Health Sciences, Nova Southeastern University, Davie, FL 33314 2, USA; dkalman@nova.edu; 3 College of Education & Human Services, Central Michigan University, Mt. Pleasant, MI 48859, USA

**Keywords:** nutrition, cognition, accuracy, processing, arginine, eSports, reaction time

## Abstract

Cognitive function is critical for successful prolonged performance in eSports. This double-blind placebo-controlled study examined the effect of an inositol-enhanced arginine silicate oral supplement on cognitive performance and energy in eSports athletes. Sixty healthy men and women who spent 5 or more hours a week playing video games were randomly assigned to take supplement or placebo for 7 days. On day 1 and 7, before and 15 min after dosing, subjects completed the Trail Making Test (TMT), Parts A and B; Stroop Test; and Profile of Mood States (POMS) questionnaire, and then played a video game for 60 min. Immediately after, cognitive tests were repeated. Self-reported energy levels increased, anger decreased, and TMT-B test errors decreased in the supplement group compared to placebo (*p* < 0.05). Fatigue, TMT-B time, and TMT B-A score improved in the supplement group compared to baseline (*p* < 0.05). After 60 min of gaming, supplementation decreased Stroop Test errors and TMT-A time (*p* < 0.05). Adverse events were minimal and not different between groups. These data appear to support the use of the studies product (nooLVL^®^) in eSports gamers looking to improve their accuracy, decision making, and reaction time during gaming.

## 1. Introduction

Inositolstabilized arginine silicate is an active nutrition ingredient (as ASI-Nitrosigine^®^) which has been shown to significantly enhance blood levels of arginine and silicon, while enhancing nitric oxide (NO) levels [1]. The product utilized in this study is a formulation of arginine silicate that has been optimized for cognitive impact through the addition of increased inositol. Inositol, also referred to as myo-inositol, is a carbocyclic sugar that is abundant in the brain and other mammalian tissues. It is known to mediate cell signal transduction in response to a variety of hormones, neurotransmitters and growth factors. Proton magnetic resonance studies show that oral administration increases inositol levels in the brain. In addition to its physiological functions in the body, inositol also aids in the manufacturing of arginine silicate as it allows for the 1:1 binding of arginine to silicon and prevents the polymerization of silicon [2]. The product studied is a novel dietary supplement composed of a specific ratio of inositol-stabilized arginine silicate and inositol that differs from ASI. The product has been validated as a nitric oxide precursor (NO) and booster [1]. NO is synthesized from the nonessential amino acid L-arginine and endogenously from the recirculation of nitrites (nitrate and nitrite) [3,4]. NO is best known for its role in vasodilation which leads to increased blood flow and cardiovascular health. However, it has been shown to be involved in many physiological processes and to affect several organ systems [5]. NO plays a role in exercise-induced vasodilation [6]. Research suggests that supplementation of L-arginine benefits hypertensive patients by correcting abnormal vasodilation. A meta-analysis of randomized, double-blind, placebo-controlled trials showed that oral L-arginine significantly lowered systolic and diastolic blood pressure compared with placebo [7]. Arginine supplementation has been shown to increase time to exhaustion during exercise [8], improve recovery [9], and offset muscular fatigue [7,10]. Active males (19–33 years of age), supplemented for 4 days with 1500 mg/day inositol-stabilized arginine silicate (ASI, Nitrosigine^®^) experienced significantly increased pre-workout energy levels, increased muscle size, and reduced biomarkers of muscle damage (i.e., CPK and LDH) during recovery from exercise [11].

The efficacy of L-arginine supplementation for cardiovascular disease [12] and exercise performance [13] is not completely agreed upon. This is most likely because of differences in bioavailability or the enzymatic breakdown of arginine. ASI complex is more effective in increasing serum arginine levels in rats than the standard arginine hydrochloride [14]. In a single-dose pharmacokinetic study in humans, ASI supplementation significantly increased the blood levels of arginine between 30 min (18.2–24.1 μg/mL) and 5 h (20.3 μg/mL) after dosing, and increased blood levels of silicon for up to 1.5 h. Furthermore, blood levels of arginine, silicon, and NO (salivary nitrite) were significantly increased after ASI supplementation for 14 days [1]. In a study to evaluate the potential pharmacokinetic differences of ASI and arginine hydrochloride (ArgHCl) at different time points, a single-dose of ASI significantly increased plasma arginine levels for up to six hours post-dose, while ArgHCl supplementation increased levels for just one hour. That indicates that ASI is a more bioavailable source of arginine [15]. In the same study, arginase levels, an enzyme that breaks down arginine, were measured. Arginase levels were significantly different between the ASI and ArgHCl groups (*p* < 0.05) after 15 days. Arginase levels were reduced in the ASI group at multiple time points after supplementation; no changes were detected in the ArgHCl group. Peaks of arginine absorption corresponded to the timepoints of low arginase levels. These data suggest that ASI reduces the enzymatic breakdown of arginine by decreasing arginase activity [16].

Silicon, a trace element, is abundant in plant-based foods and is found in over-the-counter antacids and analgesics. It enhances L-arginine bioavailability [14] and is critical for skin, hair, nails, immunity, and bone health [17,18,19]. Additionally, silicon, especially as silicic acid, may protect vascular integrity during age-related vascular diseases [19]. Silica (silicic acid) intake is associated with reduced risks of Alzheimer’s disease and dementia, which may suggest a connection between silica-intake and cognitive function [20].

NO has been tested as a potential strategy for improving mild cognitive impairment because of its role in circulation [21,22]. Especially in individuals with learning and memory impairment, the beneficial effects of NO on learning and memory are well documented [23]. While L-arginine has been shown to decrease stress and anxiety in healthy individuals [24]. It may also enhance mental focus and acuity during sporting events that require it by increasing blood flow to the brain.

Enhanced mental flexibility has been shown to benefit athletes when faced with quick decisions and associated adaptations that may be required the for optimum performance of certain sports, especially field sports. In addition, elite athletes have been shown to score higher on tests assessing mental flexibility than non-elite-athletes; therefore, enhanced mental flexibility may offer the “edge” that many athletes seek [25]. Cognitive flexibility enhances sports performance by decreasing anxiety and stress during competition [26]. Improved cognitive flexibility has been observed in team sports [27]. The ability to focus attention and anticipation, which are both part of cognitive flexibility, is heightened in the higher-level sport participant [28]. Cognitive flexibility is associated with sport success [29]. These enhancements in cognitive function may benefit several types of athletes, including gamers in in eSports.

eSports, or video game playing, is emerging as an area of research focusing on how cognitive processes, specifically executive functions, impact performance in competitive game playing. Video game playing requires task switching and rapid reactions to multiple visual and auditory cues. Experienced gamers have been shown to perform better on visual search tasks than non-experienced individuals, suggesting a role for video gaming in improving visual selective attention [30]. Research has also shown that video gaming increases cognitive flexibility [31] and that experienced video game players perform better on task switching paradigms than non-experienced individuals [30,32]. These data not only suggest that playing certain video games enhances executive functioning, but that successful performance in video gaming requires strong executive functioning. While there are some studies focused on cognitive science and player performance in eSports, research is lacking concerning treatments or methods to enhance executive function and performance in eGamers.

Several clinical studies have demonstrated that ASI has various cognitive enhancing benefits. ASI supplementation (1500 mg/day) was tested in two double-blind, placebo-controlled crossover studies using the Trail Making Test (TMT, Parts A and B) to measure cognitive function. The TMT consists of two Parts, A and B; each test is measured by the time to completion and number of errors, with lower scores indicating greater performance. TMT-A involves connecting an ascending sequence of 25 numbers, while TMT-B involves connecting an alternating sequence of 25 numbers and letters. After 4 days of supplementation, TMT-B time decreased significantly compared to placebo (33% improvement, *p* = 0.024) within a 15-min period post-dose [33]. Within 15 min after a single dose, the decrease in mean TMT B-A score, a more direct measure of cognitive flexibility, from the baseline—was significantly greater in the ASI group (−14.4 sec; −45%) compared to placebo (−1.5 sec; −4%) (*p* < 0.05 between groups) [34]. In the same study, subjects reported a significant increase in perceived energy levels after 3 days of supplementation, as measured by the Profile of Mood States (POMS) self-report questionnaire. Researchers found no significant differences in heart rate and blood pressure between groups, supporting the use of ASI as a non-stimulant energy source [35]. In a double-blind, placebo-controlled, crossover study, 24 male subjects took a single dose of ASI (1500 mg) or placebo 30 min prior to a treadmill maximally graded exercise test. Following intense exercise, time to complete TMT-A and TMT-B increased by a significant 51% and 11% respectively in the Placebo group, while it decreased by 5% for TMT-A and 7% for TMT-B when participants consumed an acute dose of ASI. These data show that ASI prevented the 51% decrease in TMT performance seen in the Placebo group following intense exercise [36].

The purpose of this investigation was to determine if the benefits of a new inositol enhanced arginine silicate transferred to e-gamers. In part, the study wanted to evaluate if a nutritional supplement could improve concentration, decrease reaction time, and increase energy levels in eGamers. This study was designed to evaluate the effectiveness of inositol enhanced arginine silicate on cognitive function, mood states, and mental performance in video gamers.

## 2. Materials and Methods

This study was a randomized, double-blind, placebo-controlled, parallel group, prospective clinical trial. The aim of this study was to evaluate the effectiveness of a new inositol enhanced arginine silicate (nooLVL^®^; Nutrition 21 Purchase, NY, USA) on cognitive performance and mood states after acute ingestion by eGamers. This study included comparing the study-product to placebo on and throughout prolonged video game playing, as well as on actual video game performance.

This study was conducted under the guidelines and principles of the Food and Drug Administration (FDA), the International Conference on Harmonization (ICH), and Good Clinical Practice (GCP). The study was executed by QPS-Missouri (Springfield, MO, USA). The study protocol and the informed consent form were reviewed by the Institutional Review Board (IRB) prior to the start of any study procedures. IRB Approval was received 13 August 2018. The IRB, Bio-Kinetic Clinical Applications Springfield, MO, USA, approved and monitored the study in accordance with the principles and requirements in 21 CFR Part 56.

### 2.1. Participants

Subjects were recruited from the greater Springfield, Missouri area. Recruitment methods included database review and local advertisements. Subjects were included if they were healthy, non-smokers, aged 18–40 years, with BMIs of 18.0 to 34.9 kg/m^2^, who played video games for 5 or more hours per week for 6 months prior to screening. In order to control for learning and familiarity, subjects agreed to supply their own operator-oriented action or strategy video game that they had played 21 times over the last 3 months, and the gaming platform with all accessories needed to play the chosen game. They agreed to continue their patterned use of the game between study visits. Baseline participant characteristics are show in Table 1.

In this study, subjects were asked to report for a total of three visits at the clinical site during the study period, including the screening visit, Visit 1, and Visit 2. They were asked to refrain from caffeine and alcohol for 12 h prior to each study visit and from any dietary supplement containing arginine for at least 7 days prior to screening and throughout the study.

After obtaining informed consent, subjects underwent screening procedures to confirm eligibility. Eligible subjects were randomized to study supplement or placebo group in a 1:1 ratio and the randomization was stratified by body mass index (BMI) of 18.0 to 27.0 and 27.1 to 34.9 kg/m^2^. Subjects were instructed to take the study supplement or placebo once daily for 7 days (+2 days). The first and the last doses were administered at the clinical site during Visit 1 and Visit 2, respectively.

### 2.2. The Product Studied

The product studied, (nooLVL^®^), and the matching placebo were provided in a blinded stick pack. The product manufacturer provided an appropriate certificate of analysis to demonstrate the dosage and safety of the test product. Subjects took 1 stick pack daily for 7 days. The subjects took the product at home except on study visit days, where they consumed the assigned product on site (witnessed dosing) in the clinic 30 min prior to the start of video game play. The studied product’s stick packs consisted of 1500 mg ASI + 100 mg of additional inositol and these inactive ingredients: citric acid, natural flavor, sucralose, acesulfame potassium, and red 40 (3.6 g each pack). The placebo contained maltodextrin, citric acid, natural flavor, sucralose, acesulfame potassium, and red 40; was matching in color, appearance, weight, etc.; was without active ingredients; and was taken once daily for 7 days. The study supplement and placebo were manufactured by Creative Flavor Concepts, Inc. under Good Manufacturing Practice (GMP) conditions and supplied by Nutrition 21, LLC (Purchase, NY, USA). The sponsor provided certificate of analyses (CoA) of the study-products for reference. Subjects were instructed to mix the stick pack with 8 oz (240 mL) of room temperature water into a shaker bottle, tightly close the top and flip-cap on the bottle and shake bottle vigorously for approximately 20 s. With the powder completely dissolved, subjects consumed the entire contents of bottle within approximately 5 min of shaking; they were instructed to swirl/shake the bottle between sips. If residue was left in the bottle, they were told to add 2 oz (60 mL) water and consume the remaining product.

### 2.3. Study Procedure

During Visit 1 (Day 1), subjects provided the study staff with the last score of their game of choice for use as the baseline game score. This was provided as a photo, screen shot, etc., but not as a verbal response. Prior to dosing, the assessments in the order of TMT-A,B, Stroop Test, and POMS were taken as the baseline. Subjects were then administered with the assigned study-product. At least 15 min after dosing, subjects were re-tested with the order of TMT-A,B, Stroop Test, and POMS. After completing the TMT-A,B, Stroop Test, and POMS, subjects started to play their chosen and pre-approved operator-oriented action or strategy games for a total of 60 min (±5 min). Immediately after completion of approximately 60 min (±5 min) of video gaming, all subjects repeated the assessments in the order of TMT-A,B and Stroop Test. If more than one round of a game was played, all game scores were recorded (in the order of playing) and the average of game scores was also recorded. Subjects were then asked several subjective questions regarding how they felt the product affected their cognitive function, game play, and energy. Subjects were then discharged from the clinical site and given the appropriate amount of the study-product with instructions on how to take the it, to last them until the seventh day (+2 days).

At Visit 2 (Day 7 (+2 days), end of study), subjects returned to the clinic. Subjects were administered with study-product on site for this end of the study. Prior to dosing, the assessments in the order of TMT-A,B, Stroop Test, and POMS were performed. At least 15 min post-dose, the assessments in the order of TMT-A,B, Stroop Test, and POMS were performed. After completing the TMT-A,B, Stroop Test, and POMS, subjects started to play for approximately 60 min (±5 min) on their chosen video game (the same game they played at Visit 1). Immediately after the completion of approximately 60 min (±5 min) of video gaming, all subjects repeated the assessments in the order of TMT-A,B, and then the Stroop Test. If more than one round of a game was played, all game scores were recorded (in the order of playing) and the average of game scores was also taken. Subjects were then asked several subjective questions regarding how they felt the study-product affected their cognitive function, game play, and energy. After these tests had been completed, subjects were assessed, and the study ended.

The safety profiles of study-products were determined by measurement of vital signs at the screening visit and the end of study visit, and by monitoring the adverse events and serious adverse events (AE/SAE) during the study period.

### 2.4. Assessments

The effectiveness of study-products (study supplement and placebo) on cognition and mood status was assessed using Trail Making Test A and B (TMT-A,B), Stroop Test, Profile of Mood States (POMS), and video game performance.

#### 2.4.1. Trail Making Test A and B (TMT-A,B)

The TMT-A,B was assessed at pre-dose, at least 15 min post-dose, and immediately after 60 min (±5 min) of video gaming at Visit 1 and Visit 2. The TMT was comprised of Parts A and B. In Part A, the subject used a pen to connect a series of 25 encircled numbers in numerical order. In Part B, the subject connected 25 encircled numbers and letters in numerical and alphabetical order, alternating between the numbers and letters. For example, the first number “1” was followed by the first letter “A” followed by the second number “2” then second letter ‘‘B’’ and so on. The numbers and letters were placed in a semi-random fixed order, in such a manner as to avoid overlapping lines being drawn by the examinee. The primary variables of interest were the total time to completion for the Parts A and B.

For scoring, total time to complete the main test, the number of errors made, and the total number of circles completed were recorded. If the subject committed 5 errors or exceeded 360 s, the test was discontinued, and the number of circles completed correctly was recorded. Part A was generally presumed to be a test of visual search and motor speed skills, whereas Part B was considered also to be a test of higher-level cognitive skills, such as mental flexibility. TMT B-A score of completion time, which was the score of the TMT-B minus TMT-A on completion time, was also analyzed. TMT B-A score provided an index of the level of interference by the addition of the flexibility component of Part B.

#### 2.4.2. Stroop Test

The Stroop Test was assessed at pre-dose, at least 15 min post-dose, and immediately after 60 min (±5 min) of video gaming during Visit 1 and Visit 2. The Stroop Test was employed to evaluate the selective attention that requires interference resolution, response inhibition, response selection, and reaction time. In the congruent test, subjects rapidly read aloud the names of colors printed in corresponding ink colors (e.g., they said, “Blue,” when the word was printed in blue). In the incongruent test, subjects rapidly read aloud the color of the ink instead of the word (e.g., they said, “Blue,” when the word “red” was printed in blue). Each test trial was timed. Scores used for analysis were the number of seconds required to complete each test. Error data were also recorded.

#### 2.4.3. Profile of Mood States (POMS)

The POMS was performed at pre-dose and at least 15 min post-dosing at Visit 1 and Visit 2. The POMS measured six subscales of mood, including tension-anxiety, depression-dejection, anger-hostility, vigor-activity, fatigue-inertia, and confusion-bewilderment. Subjects were asked to use a 5-point Likert scale (0 = not at all, 1 = a little, 2 = moderately, 3 = quite a bit, 4 = extremely), to review a list of 65 words (feelings) and rank the degree to which they were experiencing that feeling at the moment. Since high vigor-activity scores reflected good moods or emotions, whereas low scores in the other subscales reflected a good mood or emotion, the total mood disturbance (TMD) score was computed by adding the five negative subscale scores (tension-anxiety, depression-dejection, anger-hostility, fatigue-inertia, and confusion-bewilderment) and subtracting the vigor-activity score. If there was any item missing in the subscale, the score of that subscale and the TMD score were not calculated for that subject. Higher numeric values of the TMD score indicate a greater degree of mood disturbance.

#### 2.4.4. Video Game Performance

At the screening visit, the video game classification (action or strategy oriented as defined in the protocol) was confirmed by site staff.

Each subject was responsible for bringing their chosen and pre-approved video game and compatible gaming platform with accessories (excluding handheld mobile devices). Subjects must have played the chosen game at least 21 times over the past 3 months prior to screening. Subjects agreed to bring and play the same video game at Visit 1 and Visit 2 and played the chosen game as regularly played, but not in excess between study visits, to minimize changes of learning curve bias. The latest score of the chosen game prior to dosing was recorded. This was provided as a photo, screen shot, etc., but not as a verbal response.

Subject played the chosen video game for an approximately one-hour per study visit at the site. The name of chosen game and actual playing time were recorded in the case report form. Video game scores were captured per round (in the order played) and over the entire 60 min (±5 min) of play in the case report form by the staff on site.

The score at pre-dose between the screening visit and Visit 1 was recorded as baseline. Video game scores were divided into three types, including total score, rank, and time. For total score, considered a continuous variable, the video game performance was extracted by determining the change of score from baseline, and was presented by quantile of percentage. For rank and time, considered categorical variables, the video game performance was presented as an improvement by rank or time.

#### 2.4.5. Safety Assessment

The safety assessment of this study was performed by recording vital signs (blood pressure and heart rate) and continuous AE/SAE monitoring.

### 2.5. Statistical Analysis

Statistical significance was set at the 0.05 level (*p* < 0.05); when and where appropriate, 95% confidence intervals were also noted (e.g., with *t*-tests). We carried out planned comparisons that were made based on previous findings and our hypothesis [37]. The paired-*t* test or Wilcoxon sign-rank test was analyzed to compare the change from baseline within each group. A *t*-test or Wilcoxon rank-sum test was used to compare between two groups. Fisher’s exact test was utilized to compare the difference in proportions between two groups when the sample size was small; otherwise, a chi-square test was employed. A non-hierarchical statistical approach was utilized to treat each endpoint of interest separately as an independent endpoint of interest.

## 3. Results

Sixty subjects were recruited and met eligibility criteria. Thirty were randomly assigned to each group (product studied or placebo). Of those 60, 55 finished the study with at least 80% compliance, with 28 subjects in the product group and 27 in the placebo group. The mean age (mean ± SD) was 28.6 ± 5.40 years old; 50 (83.33%) subjects were male and 10 (16.67%) subjects were female.

### 3.1. TMT Part A

#### 3.1.1. Testing Visit 1

**Figure 1:**
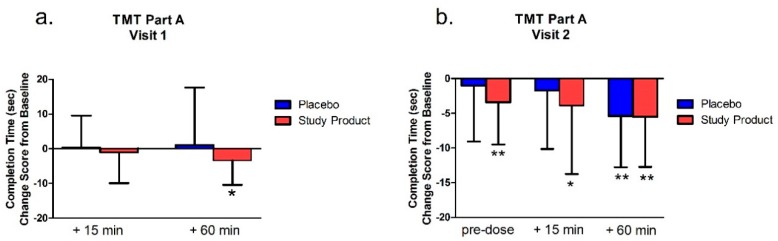
Trail Making Test Part A completion times at Visit 1 (**a**) and Visit 2 (**b**). Bar graphs represent change from Visit 1 baseline. * *p* < 0.05 versus baseline; ** *p* < 0.01 versus baseline.

As seen in Figure 1, within-group analysis of change scores from baseline for both groups (product studied and placebo) revealed that for the first testing visit (first dose of placebo or treatment), the treatment group had a significant decrease in the time to complete the task 60 min post-dose/post-gaming (*p* = 0.01). There was no change from baseline in the placebo group at the 60 min time point (*p* > 0.05). There were no significant differences between groups on latency (all *p*’s > 0.05). There were no significant differences either between or within groups in the number of errors (*p* > 0.05).

#### 3.1.2. Testing Visit 2.

As shown in Figure 1, the analyses of change scores from baseline for the second testing visit (following 7 days of daily placebo or product studied treatment) revealed that the treatment group showed a statistically significant difference from baseline at task completion pre-dose (*p* < 0.01) and at 15 min post dose at Visit 2 (*p* = 0.04). Both groups showed a significant change from baseline at 60 min post-dose/post-gaming (*p* < 0.001). For the placebo group, there was also a significant improvement in time to complete the task between pre-dose at Visit 2 and 60 min post-dose/post-gaming (*p* < 0.001). There were no significant differences between groups (*p* > 0.05) for TMT-A endpoints at Visit 2.

### 3.2. TMT Part B

#### 3.2.1. Testing Visit 1

**Figure 2:**
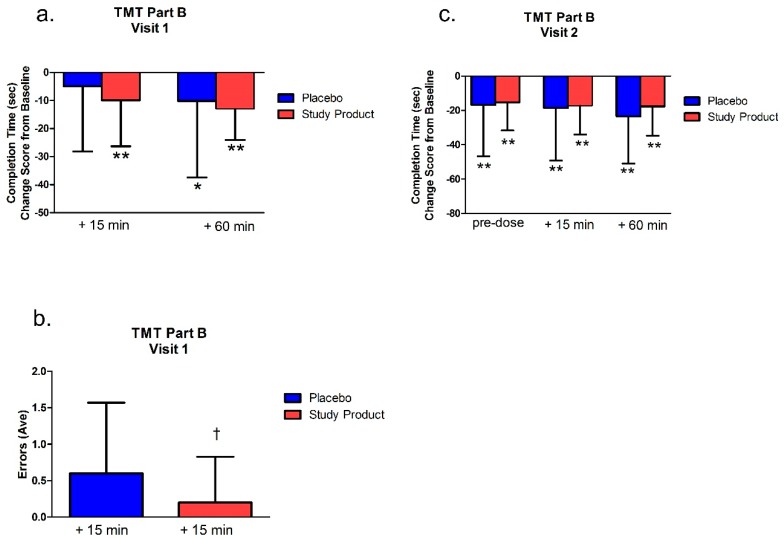
Trail Making Test Part B completion times and errors at Visit 1 (**a**,**b**) and completion times at Visit 2 (**c**). Bar graphs represent change from Visit 1 baseline. * *p* < 0.05 versus baseline; ** *p* < 0.01 versus baseline; † *p* < 0.05 versus placebo.

Within-group analysis of change scores from baseline showed that the study product group was faster at completing the TMT-B at the 15 min time point and at 60 min post-dose/post-gaming (*p* < 0.01 and *p* < 0.0001 respectively) (see Figure 2). Fifteen minutes after treatment, the study product group made significantly less errors than the placebo group (*p* = 0.04). There were no other significant differences either between or within groups in the number of errors (*p* > 0.05).

#### 3.2.2. Testing Visit 2

As shown in Figure 2, within-group analyses of change scores from baseline (following 7 days of daily product studied or placebo) showed that both groups (product studied and placebo) were faster at completing the TMT-B at all time points (pre-dose, and 15 min and 60 min post-dose/post-gaming, all *p* values < 0.0001), see Figure 2. There were no significant differences between groups in change scores from Visit 2 pre-dose scores (*p* > 0.05, data not shown), except that in the placebo group there was a significant improvement in time to complete the task between pre-dose at Visit 2 and 60 min post-dose/post-gaming (*p* < 0.01, data not shown).

### 3.3. TMT B-A Score

#### 3.3.1. Testing Visit 1

**Figure 3:**
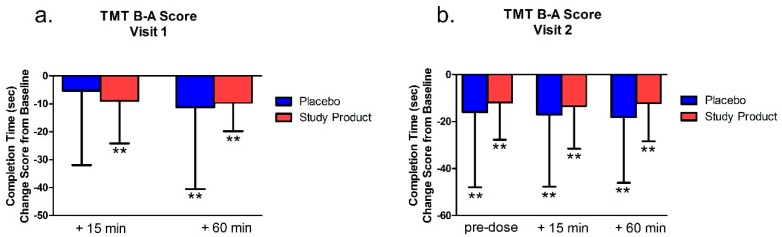
Trail Making B-A Scores at Visits 1 (**a**) and 2 (**b**). Bar graphs represent change from Visit 1 baseline. ** = *p* < 0.01 versus baseline.

As shown in Figure 3, within-group analysis for both groups (product studied and placebo) revealed that the treatment group had a significantly different TMT B-A change score at 15 min post-dose (*p* = 0.005). Both the study product groupand the placebo group were faster at 60 min post-dose/post-gaming (*p*’s < 0.01), compared to their baseline performance. While there were no significant differences between groups (*p* > 0.05), there was a statistical trend for between-group differences at the 15 min time point (*p* = 0.06).

#### 3.3.2. Testing Visit 2

Overall, The TMT B-A completion time change from baseline scores showed that both groups, at all time points, were significantly faster (*p* < 0.001), see Figure 3. There were no significant differences between groups (all *p* values > 0.05).

### 3.4. Stroop Test

#### 3.4.1. Congruent Stimuli

There were no significant differences between the groups at baseline (*p* = 0.66). Compared to baseline performance, the total time to complete the task was significantly different for both groups at 15 min and 60 min for Visit 1 (*p* < 0.01), and at pre-dose, 15 min and 60 min for Visit 2 (all *p*’s < 0.01; data not shown). This indicates overall ceiling effects for this task. There were no significant differences between groups.

#### 3.4.2. Incongruent Stimuli

##### Testing Visit 1

**Figure 4:**
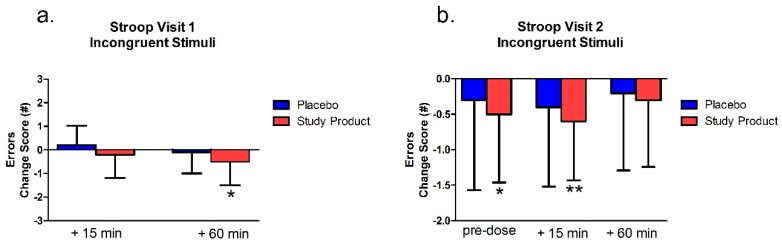
Stroop Test incongruent stimuli errors at Visits 1 (**a**) and 2 (**b**). Bar graphs represent change in errors from Visit 1 baseline. * = *p* < 0.05 versus baseline; ** = *p* < 0.01 versus baseline.

Within-group analyses of change scores compared to group baseline showed that the placebo group was significantly faster at the 15 min time point (*p* = 0.02). Though this pattern reversed, with only the study product group having a significant change score at the 60 min post-dose/post-gaming time point (*p* < 0.01; data not shown). Within-group analysis of the number of errors compared to group baseline showed that the treatment group was significantly improved at 60 min post-dose/post-gaming (*p* = 0.03; see Figure 4). There were no significant between-group differences.

##### Testing Visit 2

Within-group analyses suggested a ceiling effect for change from baseline time to complete the task. Both groups showed a significant change at all time points (*p* < 0.05; data not shown). Within-group analysis from the Visit 2 pre-dose showed a significant difference in change score for the placebo group at 60 min (*p* < 0.01; data not shown). Within group analysis of error change scores showed that the study product group made significantly fewer errors from baseline at Visit 2 pre-dose and 15 min post-dose (*p* < 0.05; see Figure 4). There were no between-group differences.

### 3.5. Profile of Mood States (POMS)

#### 3.5.1. Testing Visit 1

**Figure 5:**
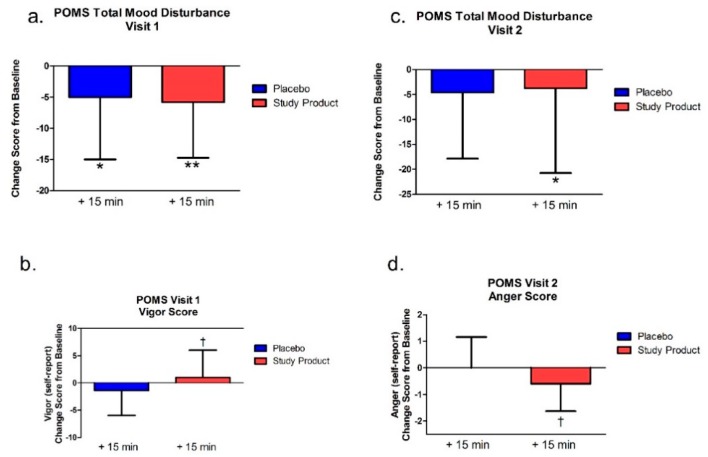
Profile of Mood States (POMS) at Visits 1 (**a**,**b**) and 2 (**c**,**d**). (**a**,**c**) show total mood disturbance (TMD) scores while (**b**,**d**) show significant subtest dimensions versus placebo. The POMS was administered at baseline and 15 min post-dose. Bar graphs represent change from visit 1 baseline. * *p* < 0.05 versus baseline; ** *p* < 0.01 versus baseline; † *p* < 0.05 versus placebo.

The POMS was only administered at baseline and at the 15 min post-dose time point. The composite (Total Mood Disturbance) change score was significant for both the study product (*p* = 0.002) and the placebo groups (*p* = 0.01; see Figure 5). Between-group analysis revealed a significant difference between groups for the subcomponent “vigor” (*p* = 0.04), where the study product group had more self-reported vigor (energy) compared to the placebo group; see Figure 5.

#### 3.5.2. Testing Visit 2

The POMS was only administered at baseline and at the 15 min post-dose time point. The composite (Total Mood Disturbance) change score was significant for the treatment (*p* = 0.01) but not the control group (*p* = 0.08) compared to baseline. Between-group analysis revealed a significant difference between groups for the subcomponent “anger” (*p* = 0.02), where the study product group had a greater decrease in self-reported anger compared to the Placebo group, see Figure 5.

### 3.6. Video Game Performance

Overall, within the groups, there were improvements in video game performance. However, there was no between group difference over the other for performance enhancement (*p* > 0.05).

## 4. Discussion

Overall, the data from this study demonstrates the utility of the product studied for supporting and enhancing cognitive function in adult-aged eGamers. The data regarding ASI as part of the product studied demonstrates that it can improve processing speed, mental acuity, and cognitive flexibility, suggesting that this supplementation would benefit eSports gamers during video game playing, a mentally exhausting task. Inositol is found at high levels in the brain and is needed for the functioning of various neurotransmitters involved in learning and memory. The current study-product, per the manufacturer, was designed to further promote the cognitive enhancing effects of the active arginine by adding an optimized dose of inositol. The aim of this study was to determine if the benefits of a new inositol enhanced arginine silicate could be transferred to e-gamers. This study tested the idea that a nutritional supplement could improve concentration, improve reaction times and increase energy levels in eGamers. This study was designed to evaluate the effectiveness of the inositol-enhanced arginine silicate (product studied: nooLVL^®^) on cognitive function, mood states, and mental performance in video gamers.

The Trail Making Test (TMT) Part A and B assesses visuomotor tracking and cognitive executive functions—especially attention. TMT-B also assesses mental flexibility. As these tests yield response latency and error scores, faster times indicate better functioning in these areas [38]. We found that in the first and second testing visits (after 7 days of treatment), the study product treatment group showed improved performance from baseline measures in the TMT Part A. Notably, however, in the TMT Part B, only the study product group showed a significant change over baseline in the 15 min post-dose time point. After this, both groups improved over baseline at all following time points in both visits. Between-group differences were also apparent at the 15 min time point where the treatment group showed an advantage over placebo in the number of errors made. Combined, these data suggest that the study product improved executive function over placebo, but that these changes, especially changes in mental flexibility (TMT Part B), are predominately acute.

The Stroop Test measures executive function—particularly the domains of selective attention and processing speed [39]. In the incongruent (more difficult) condition, the change scores for time to completion showed an advantage for the study product group at the 60 min time point in the first visit only. There was a general advantage for the study product group over placebo in the number of errors made. In the first visit, the change from baseline in the study product group showed fewer errors at the 60 min (post-gaming) time point. Following 7 days of treatment, change scores from baseline still showed less errors for the study product group at the 15 and 60 min time point. This was not the case for the placebo group. These findings suggest that, unlike TMT, the study product decreases errors in an executive function task acutely and that this advantage is sustained for at least 7 days of continued treatment.

In addition to the cognitive measures, we also assessed how the study product affects mood through an assessment of total mood disturbance. Unlike the cognitive tests, this measure was only given at baseline and 15 min after the study product at both testing visits (since the emotional valence of the video games could confound this measure at the 60 min time point). In the first visit, change scores from baseline showed that both groups had a decrease in mood disturbances; however, one week later, only the study product group had a significant decrease in total mood disturbance from baseline. Notably, compared to placebo, the study product group reported more vigor (energy) at the first testing visit and less anger at the second testing visit.

The combined neurobehavioral findings indicate that the study product is ineffective on easy cognitive tasks (the Congruent Stroop Test). Changes in reaction times and mental flexibility (TMT-B) are mostly acutely affected by treatment. There appears to be a sustained effect for decreased errors on a difficult task (Stroop Incongruent Test). Unlike the cognitive tasks, the effect of experimental treatment on mood suggests that sustained treatment is more effective for improved mood over baseline measures.

The effects of the study product on video game performance were mixed, with only some participants showing improvement. It is not certain, therefore, whether the study product had an impact on actual performance. One limitation of this study may be that we did not control for factors that may contribute to mental fatigue, such as work hours, etc. However, we did bring everyone in under standard “life” conditions. Future studies may want to consider that. Follow up studies will need to ascertain if, consistent with our neurobehavioral findings, the treatment is more effective at decreasing errors as task difficulty increases.

## 5. Conclusions

The results of this randomized, double-blind, placebo-controlled study demonstrated that the product studied improved executive functioning before and after video game playing. Enhancements were seen in processing speed, task switching, and selective attention in eGamers taking the product studied, as observed by positive changes in TMT and Stroop Test performance. Moreover, the product studied increased perceived vigor (energy) levels in eGamers after a single dose, and decreased anger. These data support the use of nooLVL^®^ in eSport gamers looking to improve their accuracy, decision making, and reaction time during video game playing.

## Figures and Tables

**Table 1:** Baseline participant characteristics.

Characteristic	Treatment		Placebo	
	*M*	*SD*	*M*	*SD*
Age	27.8	5.51	29.3	5.29
Height (cm)	178.6	6.67	177.8	9.41
Weight (kg)	86.02	17.27	87.72	15.64
BMI (kg/m^2^)	26.87	4.74	27.72	4.38
Heart Rate (bpm)	77.7	11.33	78.9	13.52
SBP (mmHg)	124.6	8.41	122.2	9.93
DBP (mmHg)	79.9	6.35	78.3	6.94
Male	27		23	
Female	3		7	

Note. BMI = body mass index; SBP = systolic blood pressure; DBP = diastolic blood pressure.
